# *N*-Methyl­serotonin hydrogen oxalate

**DOI:** 10.1107/S2414314623003784

**Published:** 2023-05-05

**Authors:** Marilyn Naeem, Nicholas A. Anas, Andrew R. Chadeayne, James A. Golen, David R. Manke

**Affiliations:** a University of Massachusetts Dartmouth, 285 Old Westport Road, North Dartmouth, MA 02747, USA; b CaaMTech, Inc., 58 East Sunset Way, Suite 209, Issaquah, WA 98027, USA; University of Antofagasta, Chile

**Keywords:** crystal structure, tryptamines, indoles, hydrogen bonding

## Abstract

The solid-state structure of *N*-methyl­serotonin {systematic name: [2-(5-hy­droxy-1*H*-indol-3-yl)eth­yl](meth­yl)aza­nium hydrogen oxalate}, C_11_H_15_N_2_O^+^·C_2_HO_4_^−^, is reported. The structure possesses a singly protonated *N*-methylserotonin cation and one hydrogen oxalate anion in the asymmetric unit. In the crystal, the mol­ecules are linked by N—H⋯O and O—H⋯O hydrogen bonds into a three-dimensional network.

## Abstract 2

The structure of the natural product *N*-methyl­serotonin is reported as its hydrogen oxalate salt.

## Graphical abstract

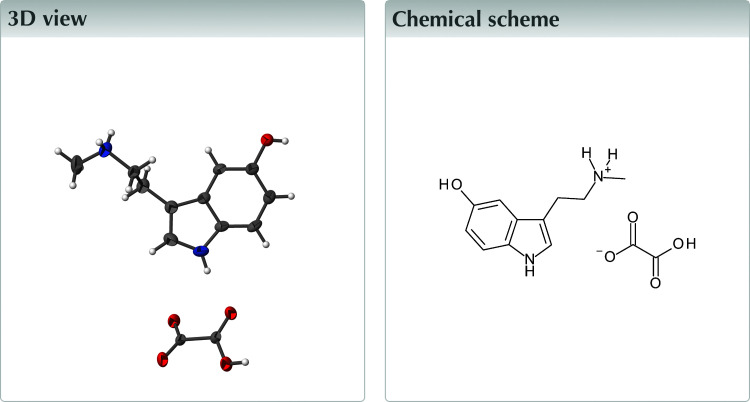

## Structure description

Serotonin (5-hy­droxy­tryptamine) is a ubiquitous neurotransmitter that is integral in regulating mood, anxiety and happiness in humans (Young & Leyton, 2002 ▸). Methyl­ating the ethyl­amine nitro­gen atom of serotonin provides three serotonin analogues: (i) *N*-methyl­serotonin, (ii) 5-hy­droxy-*N*,*N*-di­methyl­tryptamine (bufotenine) and (iii) 5-hy­droxy-*N*,*N*,*N*-tri­methyl­tryptammonium (bufotenidine). Of these, bufotenine is probably most widely known as a natural product found in the secretions of *Bufo alvarius* toads. Bufotenine is a potent agonist of serotonin receptors and is one of several compounds to which the psychedelic effects of toad secretions are attributed (Egan *et al.*, 2000 ▸).

Replacing three hydrogen atoms with methyl groups in the ethyl­amine group of serotonin provides 5-hy­droxy-*N*,*N*,*N*-tri­methyl­tryptammonium, or bufotenidine, which is also a natural product found in toad secretions. Bufotenidine differs from the other analogues by virtue of its quaternary ammonium cation and selective affinity for the serotonin 3 receptor. Due to its charge, bufotenidine is unable to cross the blood–brain barrier, restricting its activity to the periphery, where it has been shown to have paralytic properties (Bhattacharya & Sanyal, 1972 ▸).

The title compound is the mono-methyl­ated variant 5-hy­droxy-*N*-methyl­tryptamine, which is a naturally occurring derivative of serotonin that has garnered attention due to its potential applications in biological and medical contexts. Endogenous *N*-methyl­serotonin has been observed both in plants and mammals, including in rodents colonized with human gut bacterial strains (Han *et al.*, 2022 ▸). The biosynthesis of *N*-methyl­serotonin most likely occurs *via N*-methyl­ation of serotonin by the enzyme indole­thyl­amine-*N*-methyl­transferase (Thompson *et al.*, 2001 ▸). This enzyme, originally discovered as the enzyme responsible for the synthesis of the endogenous hallucinogen di­methyl­tryptamine (Barker *et al.*, 2012 ▸), has recently been shown to have a broader substrate scope, including serotonin, which likely leads to the formation of *N*-methyl­serotonin (Chu *et al.*, 2014 ▸).

The pharmacological properties of *N*-methyl­serotonin have been a subject of increasing inter­est. It is reported to have significant binding affinity for the serotonin 1 A and 7 receptors, in addition to being a potent serotonin reuptake inhibitor (Powell *et al.*, 2008 ▸). These activities suggest that *N*-methyl­serotonin may have a unique pharmacological profile different from parent serotonin and may provide novel therapeutic opportunities for various psychiatric and neurological disorders. The title compound was first synthesized by Hofmann in 1955 and characterized by IR and elemental analysis (Stoll *et al.*, 1955 ▸). Herein, the crystal structure of 5-hy­droxy-*N*-methyl­tryptamine is presented as its hydrogen oxalate salt.

**Figure 1:**
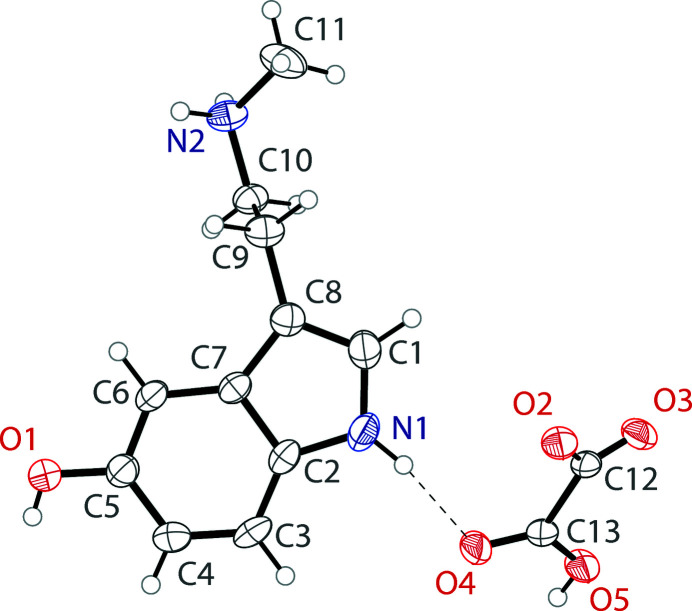
The mol­ecular structure of 5-hy­droxy-*N*-methyl­tryptammonium hydrogen oxalate showing the atomic labeling. Displacement ellipsoids are drawn at the 50% probability level. Hydrogen bonds are shown as dashed lines.

**Figure 2:**
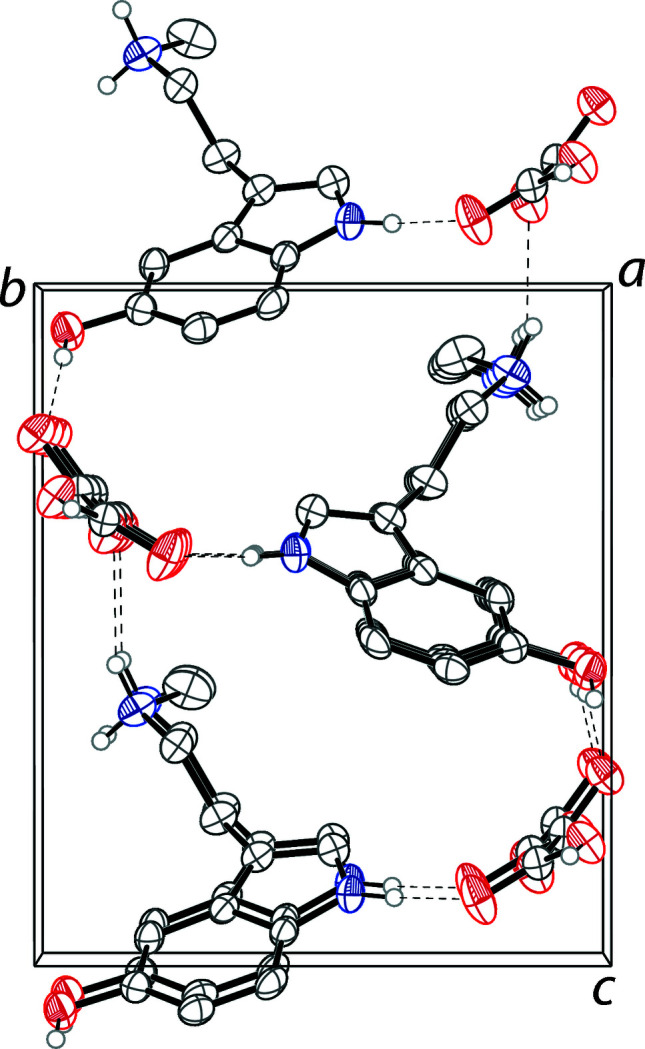
The crystal packing of 5-hy­droxy-*N*-methyl­tryptammonium hydrogen oxalate shown along the *a*-axis. Hydrogen bonds are shown as dashed lines. H atoms not involved in hydrogen bonding are omitted for clarity.

The asymmetric unit of 5-hy­droxy-*N*-methyl­trypt­ammonium hydrogen oxalate contains one tryptammonium cation and one hydrogen oxalate anion (Fig. 1 ▸). The trypt­ammonium cation has a near planar indole unit with an r.m.s. deviation from planarity of 0.014 Å. The ethyl­amino arm is turned away from the indole plane with a C7—C8—C9—C10 torsion angle of −83.1 (3)°. The *N*-methyl group of this arm possesses a *gauche* configuration , with a C9—C10—N2—C11 torsion angle of 57.2 (3)°. The hydrogen oxalate anion varies significantly from planarity, with a CO_2_-to-CO_2_ plane-to-plane twist angle of 24.2 (1)°. The ions are linked together through a series of N—H⋯O and O—H⋯O hydrogen bonds into a three-dimensional framework (Fig. 2 ▸, Table 1 ▸). The hydrogen oxalate ions are linked together through O—H⋯O hydrogen bonds into chains along (100).

The most closely related mono­alkyl­tryptamine structure to the title compound is 5-meth­oxy-*N*-methyl­tryptamine [Cambridge Structural Database (Groom *et al.*, 2016 ▸) refcode QQQAHA; Bergin *et al.*, 1968 ▸]. There are six other mono­alkyl­tryptamine structures reported in the literature. These are the natural product norpsilocin, 4-hy­droxy-*N*-methyl­tryptamine, which has been reported as its free base and its fumarate salt (MULXAV and MULXEZ; Chadeayne *et al.*, 2020 ▸), the natural product baeocystin (FEJBAB; Naeem *et al.*, 2022*b* ▸), 4-acet­oxy-*N*-methyl­tryptamine (Glatfelter *et al.*, 2022 ▸), 4-benz­yloxy-*N*-iso­propyl­tryptammonium chloride and 4-hy­droxy-*N*-iso­propyl­tryptamine (CCDC 2246619 and 2246620; Laban *et al.*, 2023 ▸). The 5-hy­droxy­tryptamine structures that are known include the natural products serotonin (JECDII; Naeem *et al.*, 2022*a* ▸), bufotenine (BUFTEN; Falkenberg, 1972 ▸) and bufotenidine (ILUVET; Pham *et al.*, 2021 ▸). The structure of serotonin has also been determined as its hydrogen oxalate salt (SERHOX: Amit *et al.*, 1978 ▸).

## Synthesis and crystallization

Single crystals suitable for X-ray diffraction studies were grown from an aqueous solution of a commercial sample (Sigma-Aldrich).

## Refinement

Crystal data, data collection and structure refinement details are summarized in Table 2 ▸.

## Supplementary Material

Crystal structure: contains datablock(s) I. DOI: 10.1107/S2414314623003784/bx4024sup1.cif

Structure factors: contains datablock(s) I. DOI: 10.1107/S2414314623003784/bx4024Isup2.hkl

Supporting information file. DOI: 10.1107/S2414314623003784/bx4024Isup3.cml

CCDC reference: 2259219

Additional supporting information:  crystallographic information; 3D view; checkCIF report

## Figures and Tables

**Table 1:** Hydrogen-bond geometry (Å, °)

*D*—H⋯*A*	*D*—H	H⋯*A*	*D*⋯*A*	*D*—H⋯*A*
N1—H1*A*⋯O4	0.87 (1)	2.08 (2)	2.928 (3)	164 (3)
N2—H2*A*⋯O1^i^	0.91 (1)	2.30 (3)	2.862 (3)	120 (2)
N2—H2*A*⋯O2^ii^	0.91 (1)	2.35 (2)	3.150 (3)	147 (3)
N2—H2*B*⋯O3^iii^	0.90 (1)	2.15 (2)	2.906 (3)	141 (3)
N2—H2*B*⋯O5^iii^	0.90 (1)	2.34 (2)	3.117 (3)	145 (3)
O1—H1⋯O3^iv^	0.77 (4)	2.00 (4)	2.768 (2)	172 (4)
O5—H5⋯O2^v^	0.84 (4)	1.76 (4)	2.595 (2)	177 (4)

Symmetry codes: (i) 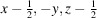 ; (ii) 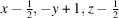 ; (iii) 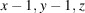 ; (iv) 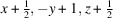 ; (v) 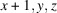 .

**Table 2:** Experimental details

Crystal data	
Chemical formula	C_11_H_15_N_2_O^+^·C_2_HO_4_^−^
*M*_r_	280.28
Crystal system, space group	Monoclinic, *P* *n*
Temperature (K)	300
*a*, *b*, *c* (Å)	5.7044 (4), 9.9485 (7), 11.7687 (7)
β (°)	90.321 (2)
*V* (Å^3^)	667.87 (8)
*Z*	2
Radiation type	Mo *K*α
μ (mm^−1^)	0.11
Crystal size (mm)	0.30 × 0.22 × 0.06

Data collection	
Diffractometer	Bruker D8 Venture CMOS
Absorption correction	Multi-scan (*SADABS*; Krause *et al.*, 2015 ▸)
*T*_min_, *T*_max_	0.715, 0.745
No. of measured, independent and observed [*I* > 2σ(*I*)] reflections	29160, 2730, 2670
*R*_int_	0.032
(sin θ/λ)_max_ (Å^−1^)	0.626

Refinement	
*R*[*F* ^2^ > 2σ(*F* ^2^)], *wR*(*F* ^2^), *S*	0.030, 0.077, 1.10
No. of reflections	2730
No. of parameters	202
No. of restraints	5
H-atom treatment	H atoms treated by a mixture of independent and constrained refinement
Δρ_max_, Δρ_min_ (e Å^−3^)	0.12, −0.21
Absolute structure	Flack *x* determined using 1280 quotients [(*I* ^+^)−(*I* ^−^)]/[(*I* ^+^)+(*I* ^−^)] (Parsons *et al.*, 2013 ▸)
Absolute structure parameter	−0.2 (2)

Computer programs: *APEX4* and *SAINT* (Bruker, 2021 ▸), *SHELXT2014* (Sheldrick 2015*a* ▸), *SHELXL2018* (Sheldrick, 2015*b* ▸), *OLEX2* (Dolomanov *et al.*, 2009 ▸) and *publCIF* (Westrip, 2010 ▸).

## full crystallographic data

### Crystal data

**Table:**

C_11_H_15_N_2_O^+^·C_2_HO_4_^−^	*F*(000) = 296
*M**_r_* = 280.28	*D*_x_ = 1.394 Mg m^−^^3^
Monoclinic, *P**n*	Mo *K*α radiation, λ = 0.71073 Å
*a* = 5.7044 (4) Å	Cell parameters from 9870 reflections
*b* = 9.9485 (7) Å	θ = 2.7–26.4°
*c* = 11.7687 (7) Å	µ = 0.11 mm^−^^1^
β = 90.321 (2)°	*T* = 300 K
*V* = 667.87 (8) Å^3^	Block, brown
*Z* = 2	0.30 × 0.22 × 0.06 mm

### Data collection

**Table:**

Bruker D8 Venture CMOS diffractometer	2670 reflections with *I* > 2σ(*I*)
φ and ω scans	*R*_int_ = 0.032
Absorption correction: multi-scan (SADABS; Krause *et al.*, 2015)	θ_max_ = 26.4°, θ_min_ = 3.5°
*T*_min_ = 0.715, *T*_max_ = 0.745	*h* = −7→7
29160 measured reflections	*k* = −12→12
2730 independent reflections	*l* = −14→14

### Refinement

**Table:**

Refinement on *F*^2^	Hydrogen site location: mixed
Least-squares matrix: full	H atoms treated by a mixture of independent and constrained refinement
*R*[*F*^2^ > 2σ(*F*^2^)] = 0.030	*w* = 1/[σ^2^(*F*_o_^2^) + (0.0456*P*)^2^ + 0.0849*P*] where *P* = (*F*_o_^2^ + 2*F*_c_^2^)/3
*wR*(*F*^2^) = 0.077	(Δ/σ)_max_ < 0.001
*S* = 1.10	Δρ_max_ = 0.12 e Å^−^^3^
2730 reflections	Δρ_min_ = −0.21 e Å^−^^3^
202 parameters	Absolute structure: Flack *x* determined using 1280 quotients [(*I*^+^)-(*I*^-^)]/[(*I*^+^)+(*I*^-^)] (Parsons *et al.*, 2013)
5 restraints	Absolute structure parameter: −0.2 (2)

### Special details

**Table:**

Geometry. All esds (except the esd in the dihedral angle between two l.s. planes) are estimated using the full covariance matrix. The cell esds are taken into account individually in the estimation of esds in distances, angles and torsion angles; correlations between esds in cell parameters are only used when they are defined by crystal symmetry. An approximate (isotropic) treatment of cell esds is used for estimating esds involving l.s. planes.
Refinement. Hydrogen atoms H1, H1A, H2A, H2B and H5 were found in a difference-Fourier map. These H atoms were refined isotropically, using *DFIX* restraints with *N*–*H*(indole) distances of 0.87 (1) Å and *N*–*H*(ammonium) distances of 0.90 (1) Å. Isotropic displacement parameters were set to 1.2*U*_eq_ of the parent nitrogen atoms and 1.5*U*_eq_ of the parent oxygen atoms. All other H atoms were placed in calculated positions [C—H = 0.93 Å (*sp*^2^), 0.97 Å (CH_2_), 0.96 Å (CH_3_)].

### Fractional atomic coordinates and isotropic or equivalent isotropic displacement parameters (Å^2^)

**Table:**

	*x*	*y*	*z*	*U*_iso_*/*U*_eq_	
O1	0.6078 (3)	0.04614 (17)	0.56240 (16)	0.0420 (4)	
N1	0.4085 (4)	0.5480 (2)	0.39536 (18)	0.0393 (5)	
N2	−0.1882 (3)	0.1720 (2)	0.12931 (17)	0.0349 (4)	
C1	0.2113 (5)	0.5182 (2)	0.3334 (2)	0.0380 (5)	
H1B	0.127417	0.579809	0.289859	0.046*	
C2	0.4845 (4)	0.4328 (2)	0.44898 (18)	0.0300 (4)	
C3	0.6748 (4)	0.4108 (2)	0.5213 (2)	0.0354 (5)	
H3	0.772969	0.480992	0.543043	0.043*	
C4	0.7131 (4)	0.2820 (2)	0.55953 (19)	0.0349 (5)	
H4	0.840919	0.264578	0.606681	0.042*	
C5	0.5629 (4)	0.1764 (2)	0.52871 (19)	0.0324 (5)	
C6	0.3690 (4)	0.1982 (2)	0.46036 (19)	0.0312 (4)	
H6	0.267239	0.128191	0.442551	0.037*	
C7	0.3287 (4)	0.3280 (2)	0.41840 (18)	0.0286 (4)	
C8	0.1551 (4)	0.3856 (2)	0.34432 (18)	0.0322 (5)	
C9	−0.0508 (4)	0.3163 (3)	0.29002 (19)	0.0357 (5)	
H9A	−0.170818	0.382400	0.272639	0.043*	
H9B	−0.116215	0.252428	0.343399	0.043*	
C10	0.0157 (4)	0.2435 (2)	0.18230 (19)	0.0307 (4)	
H10A	0.078092	0.307744	0.128362	0.037*	
H10B	0.138068	0.178790	0.199422	0.037*	
C11	−0.3888 (4)	0.2595 (3)	0.1022 (2)	0.0506 (7)	
H11A	−0.495409	0.212713	0.052715	0.076*	
H11B	−0.333959	0.339444	0.065125	0.076*	
H11C	−0.468021	0.283580	0.171023	0.076*	
C12	0.4759 (3)	0.9157 (2)	0.30591 (18)	0.0279 (4)	
C13	0.7233 (4)	0.8699 (2)	0.34523 (19)	0.0291 (4)	
O2	0.3103 (3)	0.86964 (18)	0.36400 (16)	0.0392 (4)	
O3	0.4636 (3)	0.98887 (17)	0.22060 (15)	0.0390 (4)	
O4	0.7505 (3)	0.76747 (19)	0.39757 (19)	0.0493 (5)	
O5	0.8902 (3)	0.95034 (18)	0.31279 (16)	0.0389 (4)	
H1A	0.487 (5)	0.623 (2)	0.399 (3)	0.054 (9)*	
H2A	−0.142 (5)	0.135 (3)	0.0625 (17)	0.046 (8)*	
H2B	−0.234 (6)	0.107 (2)	0.177 (2)	0.048 (8)*	
H1	0.715 (7)	0.040 (3)	0.602 (3)	0.054 (10)*	
H5	1.024 (7)	0.922 (4)	0.330 (3)	0.067 (11)*	

### Atomic displacement parameters (Å^2^)

**Table:**

	*U*^11^	*U*^22^	*U*^33^	*U*^12^	*U*^13^	*U*^23^
O1	0.0454 (10)	0.0325 (9)	0.0478 (10)	−0.0060 (8)	−0.0167 (8)	0.0050 (7)
N1	0.0501 (12)	0.0251 (9)	0.0427 (11)	−0.0081 (9)	0.0002 (9)	−0.0014 (8)
N2	0.0249 (9)	0.0442 (11)	0.0355 (10)	−0.0037 (8)	0.0011 (7)	−0.0082 (9)
C1	0.0485 (13)	0.0315 (11)	0.0341 (11)	0.0033 (10)	−0.0005 (9)	0.0013 (9)
C2	0.0357 (11)	0.0251 (9)	0.0292 (10)	−0.0062 (9)	0.0059 (8)	−0.0047 (8)
C3	0.0379 (12)	0.0342 (11)	0.0342 (11)	−0.0142 (9)	0.0012 (9)	−0.0113 (9)
C4	0.0338 (11)	0.0405 (12)	0.0304 (10)	−0.0041 (9)	−0.0042 (8)	−0.0043 (9)
C5	0.0354 (11)	0.0303 (11)	0.0314 (10)	−0.0034 (9)	−0.0001 (8)	−0.0009 (8)
C6	0.0325 (11)	0.0268 (10)	0.0344 (11)	−0.0084 (9)	−0.0008 (8)	−0.0027 (8)
C7	0.0298 (10)	0.0276 (10)	0.0282 (9)	−0.0035 (8)	0.0028 (8)	−0.0054 (8)
C8	0.0347 (11)	0.0329 (11)	0.0292 (10)	0.0007 (9)	0.0010 (8)	−0.0043 (9)
C9	0.0301 (11)	0.0427 (12)	0.0344 (11)	−0.0006 (9)	0.0001 (9)	−0.0059 (9)
C10	0.0223 (9)	0.0335 (10)	0.0364 (11)	−0.0011 (8)	0.0000 (8)	−0.0040 (8)
C11	0.0285 (12)	0.074 (2)	0.0487 (15)	0.0014 (12)	−0.0078 (10)	0.0122 (13)
C12	0.0192 (9)	0.0255 (9)	0.0389 (11)	−0.0006 (7)	−0.0053 (8)	−0.0010 (9)
C13	0.0215 (9)	0.0296 (10)	0.0361 (10)	−0.0024 (8)	−0.0052 (7)	0.0010 (9)
O2	0.0187 (7)	0.0423 (9)	0.0567 (10)	−0.0034 (6)	−0.0011 (7)	0.0103 (8)
O3	0.0264 (7)	0.0443 (9)	0.0463 (9)	−0.0007 (7)	−0.0098 (6)	0.0118 (8)
O4	0.0290 (8)	0.0397 (10)	0.0790 (13)	−0.0060 (7)	−0.0172 (8)	0.0244 (9)
O5	0.0170 (7)	0.0445 (10)	0.0554 (10)	−0.0019 (6)	−0.0011 (7)	0.0172 (8)

### Geometric parameters (Å, º)

**Table:**

O1—C5	1.378 (3)	C6—H6	0.9300
O1—H1	0.77 (4)	C6—C7	1.401 (3)
N1—C1	1.370 (3)	C7—C8	1.435 (3)
N1—C2	1.377 (3)	C8—C9	1.501 (3)
N1—H1A	0.873 (14)	C9—H9A	0.9700
N2—C10	1.496 (3)	C9—H9B	0.9700
N2—C11	1.472 (3)	C9—C10	1.510 (3)
N2—H2A	0.910 (14)	C10—H10A	0.9700
N2—H2B	0.898 (14)	C10—H10B	0.9700
C1—H1B	0.9300	C11—H11A	0.9600
C1—C8	1.363 (3)	C11—H11B	0.9600
C2—C3	1.393 (3)	C11—H11C	0.9600
C2—C7	1.415 (3)	C12—C13	1.551 (3)
C3—H3	0.9300	C12—O2	1.256 (3)
C3—C4	1.375 (4)	C12—O3	1.242 (3)
C4—H4	0.9300	C13—O4	1.200 (3)
C4—C5	1.402 (3)	C13—O5	1.303 (3)
C5—C6	1.381 (3)	O5—H5	0.84 (4)
			
C5—O1—H1	113 (3)	C6—C7—C8	133.9 (2)
C1—N1—C2	108.64 (19)	C1—C8—C7	106.3 (2)
C1—N1—H1A	129 (2)	C1—C8—C9	126.0 (2)
C2—N1—H1A	122 (2)	C7—C8—C9	127.6 (2)
C10—N2—H2A	109 (2)	C8—C9—H9A	109.2
C10—N2—H2B	108 (2)	C8—C9—H9B	109.2
C11—N2—C10	114.2 (2)	C8—C9—C10	112.22 (18)
C11—N2—H2A	106.4 (19)	H9A—C9—H9B	107.9
C11—N2—H2B	109 (2)	C10—C9—H9A	109.2
H2A—N2—H2B	110 (3)	C10—C9—H9B	109.2
N1—C1—H1B	124.7	N2—C10—C9	112.32 (17)
C8—C1—N1	110.6 (2)	N2—C10—H10A	109.1
C8—C1—H1B	124.7	N2—C10—H10B	109.1
N1—C2—C3	130.8 (2)	C9—C10—H10A	109.1
N1—C2—C7	107.5 (2)	C9—C10—H10B	109.1
C3—C2—C7	121.7 (2)	H10A—C10—H10B	107.9
C2—C3—H3	121.0	N2—C11—H11A	109.5
C4—C3—C2	117.9 (2)	N2—C11—H11B	109.5
C4—C3—H3	121.0	N2—C11—H11C	109.5
C3—C4—H4	119.4	H11A—C11—H11B	109.5
C3—C4—C5	121.2 (2)	H11A—C11—H11C	109.5
C5—C4—H4	119.4	H11B—C11—H11C	109.5
O1—C5—C4	121.2 (2)	O2—C12—C13	114.59 (18)
O1—C5—C6	117.5 (2)	O3—C12—C13	117.46 (18)
C6—C5—C4	121.3 (2)	O3—C12—O2	127.91 (19)
C5—C6—H6	120.7	O4—C13—C12	121.18 (19)
C5—C6—C7	118.7 (2)	O4—C13—O5	125.39 (19)
C7—C6—H6	120.7	O5—C13—C12	113.40 (18)
C2—C7—C8	106.89 (19)	C13—O5—H5	113 (3)
C6—C7—C2	119.2 (2)		
			
O1—C5—C6—C7	−175.6 (2)	C3—C4—C5—O1	176.8 (2)
N1—C1—C8—C7	0.1 (3)	C3—C4—C5—C6	−1.1 (3)
N1—C1—C8—C9	179.3 (2)	C4—C5—C6—C7	2.3 (3)
N1—C2—C3—C4	−177.7 (2)	C5—C6—C7—C2	−1.2 (3)
N1—C2—C7—C6	178.9 (2)	C5—C6—C7—C8	177.6 (2)
N1—C2—C7—C8	−0.2 (2)	C6—C7—C8—C1	−178.9 (3)
C1—N1—C2—C3	−179.7 (2)	C6—C7—C8—C9	1.9 (4)
C1—N1—C2—C7	0.3 (3)	C7—C2—C3—C4	2.3 (3)
C1—C8—C9—C10	97.9 (3)	C7—C8—C9—C10	−83.1 (3)
C2—N1—C1—C8	−0.2 (3)	C8—C9—C10—N2	178.81 (19)
C2—C3—C4—C5	−1.3 (3)	C11—N2—C10—C9	57.2 (3)
C2—C7—C8—C1	0.1 (2)	O2—C12—C13—O4	24.3 (3)
C2—C7—C8—C9	−179.1 (2)	O2—C12—C13—O5	−157.6 (2)
C3—C2—C7—C6	−1.1 (3)	O3—C12—C13—O4	−153.7 (2)
C3—C2—C7—C8	179.8 (2)	O3—C12—C13—O5	24.4 (3)

### Hydrogen-bond geometry (Å, º)

**Table:**

*D*—H···*A*	*D*—H	H···*A*	*D*···*A*	*D*—H···*A*
N1—H1*A*···O4	0.87 (1)	2.08 (2)	2.928 (3)	164 (3)
N2—H2*A*···O1^i^	0.91 (1)	2.30 (3)	2.862 (3)	120 (2)
N2—H2*A*···O2^ii^	0.91 (1)	2.35 (2)	3.150 (3)	147 (3)
N2—H2*B*···O3^iii^	0.90 (1)	2.15 (2)	2.906 (3)	141 (3)
N2—H2*B*···O5^iii^	0.90 (1)	2.34 (2)	3.117 (3)	145 (3)
O1—H1···O3^iv^	0.77 (4)	2.00 (4)	2.768 (2)	172 (4)
O5—H5···O2^v^	0.84 (4)	1.76 (4)	2.595 (2)	177 (4)

Symmetry codes: (i) *x*−1/2, −*y*, *z*−1/2; (ii) *x*−1/2, −*y*+1, *z*−1/2; (iii) *x*−1, *y*−1, *z*; (iv) *x*+1/2, −*y*+1, *z*+1/2; (v) *x*+1, *y*, *z*.
